# Interferome profiling in the blood links IFNα5-Mucin-1 axis to HIV-1 pathogenesis

**DOI:** 10.1128/jvi.01089-26

**Published:** 2026-09-25

**Authors:** Kejun Guo, Stephanie M. Dillon, Emily C. Meneses, Bradley S. Barrett, Alexandra Norman, Kaylee L. Mickens, James H. Morrison, Ulf Dittmer, Kathrin Sutter, Eric M. Poeschla, Katerina Kechris, Cara C. Wilson, Mario L. Santiago

**Affiliations:** 1 Division of Infectious Diseases, University of Colorado School of Medicine 548660 , Aurora, Colorado, USA; 2 RNA Bioscience Initiative, University of Colorado School of Medicine 548660 , Aurora, Colorado, USA; 3 Center for Innovative Design and Analysis, Department of Biostatistics and Informatics, University of Colorado School of Medicine 548660 , Aurora, Colorado, USA; 4 Department of Immunology and Microbiology, University of Colorado School of Medicine 548660 , Aurora, Colorado, USA; 5 Institute for Virology, University Hospital Essen, University of Duisburg-Essen 678704, Essen, Germany; 6 Institute for the Research on HIV and AIDS-associated Diseases, University Hospital Essen, University of Duisburg-Essen 119884, Essen, Germany; Icahn School of Medicine at Mount Sinai, New York, New York, USA

**Keywords:** innate immunity, HIV-1 pathogenesis, T-cell exhaustion, Mucin-1, chronic inflammation, HIV-1, interferon-alpha subtypes, interferon

## Abstract

Type I interferons (IFN-Is) are essential for antiviral immunity, but contribute to immune dysfunction during chronic HIV-1 infection. IFN-Is comprise multiple IFNα subtypes that signal through a common receptor yet exhibit distinct biological activities, but their qualitative roles in HIV-1 pathogenesis remain incompletely defined. We previously identified IFNα5 as the dominant IFN-I in the systemic circulation of untreated people with HIV (PWH). Here, we delineated IFNα subtype-specific interferomes in peripheral blood mononuclear cells (PBMCs) from HIV-uninfected donors by transcriptomic profiling, and integrated these signatures with blood transcriptomes from untreated PWH, and matched controls. Although the IFN-Is tested induced canonical interferon-stimulated genes (ISGs) *in vitro*, they also induced different transcriptional programs. *In vivo*, dysregulation of multiple canonical ISGs, including *CD38*, *LAG3*, and *CMPK2*, correlated with plasma HIV-1 RNA levels, CD4 T-cell depletion, and/or systemic inflammatory markers. By contrast, the IFN-downregulated genes *CXCL5* and *VSIG4* were repressed in PWH and inversely correlated with viral load. Notably, *MUC1*, a non-canonical ISG that was most strongly induced by IFNα5, was significantly upregulated in PWH and associated with the inhibitory receptor *LAG3*, and markers of IL-6-driven inflammation. Flow cytometric analysis demonstrated increased Mucin-1 surface density on circulating CD4 T cells of PWH vs controls. Together, these findings demonstrate that IFNα subtypes can drive qualitatively distinct responses in bulk PBMC, identify IFNα5-associated gene programs in blood that may contribute to immune dysregulation among untreated PWH, and refine current models of IFN-I involvement in HIV-1 pathogenesis.

**IMPORTANCE:**

People living with HIV experience persistent immune activation and inflammation that contribute to immune system damage and long-term health complications. Molecules known as type I interferons (IFN-Is) help control viruses early in infection, but prolonged exposure to these signals may also worsen immune dysfunction. Here, we show that IFNα5 is the dominant interferon present in the blood of people with untreated HIV and activates a distinct gene program in circulating immune cells. This program includes genes linked to immune activation, metabolic dysfunction, and immune exhaustion. IFNα5 strongly induces Mucin-1 on CD4 T cells, which is associated with systemic inflammation. These findings identify IFN pathways that may contribute to immune dysfunction during chronic HIV infection and highlight potential targets for restoring immune health.

## INTRODUCTION

Chronic HIV-1 infection is associated with inflammation, potentially contributing to severe co-morbidities (1, 2). Inflammation during HIV-1 infection has been linked to the production of cytokines that include IL-6 and TNFα (3, 4). While typically associated with antiviral responses, type I interferon (IFN-I) responses are dysregulated in persons with chronic HIV-1 infection (PWH) and could also contribute to inflammation (5, 6). However, IFN-Is are quite diverse, with the 13 IFNα subtypes and IFNβ being predominant in the systemic circulation *in vivo* (7, 8). To date, it remains unclear which of these IFN-Is are involved in the inflammatory process.

Although all IFN-Is bind to the type I IFN receptor (IFNΑR) to stimulate the JAK/STAT pathway, differences in IFNΑR binding affinities result in *quantitative* differences in interferon-stimulated gene (ISG) induction (9, 10). Earlier studies revealed that stimulating cells with similar IFN-I protein levels *in vitro* results in lower canonical ISG induction (such as *ISG15*) for IFNα subtypes with lower binding affinity to IFNAR, particularly IFNα1 (11). Interestingly, even after normalizing for quantitative differences in canonical ISG induction, the genes altered by diverse IFNα subtypes and IFNβ do not completely overlap. In gut CD4 T cells, IFNβ stimulated 2.3-fold more genes than individual IFNα subtypes. IFNα5 and IFNα14 altered over 200 genes not detected with IFNα1, IFNα2, IFNα8, IFNα14, and IFNβ (12). In human alveolar epithelial cells, IFNα5 and IFNα14 altered a significantly higher number of genes than the other IFNs tested (13). Altogether, these data, along with other observations (14), implicated *qualitative* differences between the IFNα subtypes and IFNβ.

The qualitative differences between the IFNα subtypes and IFNβ were further highlighted by studying mucosal inflammation in PWH. In the gut, a major site for HIV-1 replication and CD4 T-cell death (15–17), IFNβ was upregulated, whereas diverse IFNα subtypes were downregulated among PWH compared to age/sex-matched controls (18). IFNβ imprinted a distinctive effect in the gut compartment, as IFNβ-specific genes linked to IL-6 and TGFβ signaling were downregulated in the colon biopsies of PWH relative to controls (12).

Interestingly, a different IFN-I phenotype was observed in the blood. As other groups reported (19), IFNβ was rarely detected in blood samples from PWH, but IFNα was upregulated (18). Notably, the IFNα response was dominated by only a few subtypes, such as *IFNA5* (20). Using a next-generation sequencing (NGS)-based IFNα-subtype quantification method (21), we found that *IFNA1*, *IFNA2*, *IFNA4*, *IFNA5*, and *IFNA8* were significantly induced in blood samples from PWH compared to controls (18). At this time, it remains unknown if these IFNα subtypes stimulate distinct gene programs in PWH. We thus evaluated the genes induced by these IFNα subtypes in peripheral blood mononuclear cells (PBMCs) *in vitro*, and tracked the defined IFNα subtype “interferomes” *in vivo*, using PBMC samples from PWH and matched controls.

## RESULTS

### IFNα subtypes induce distinct interferomes in PBMCs

**Fig 1:**
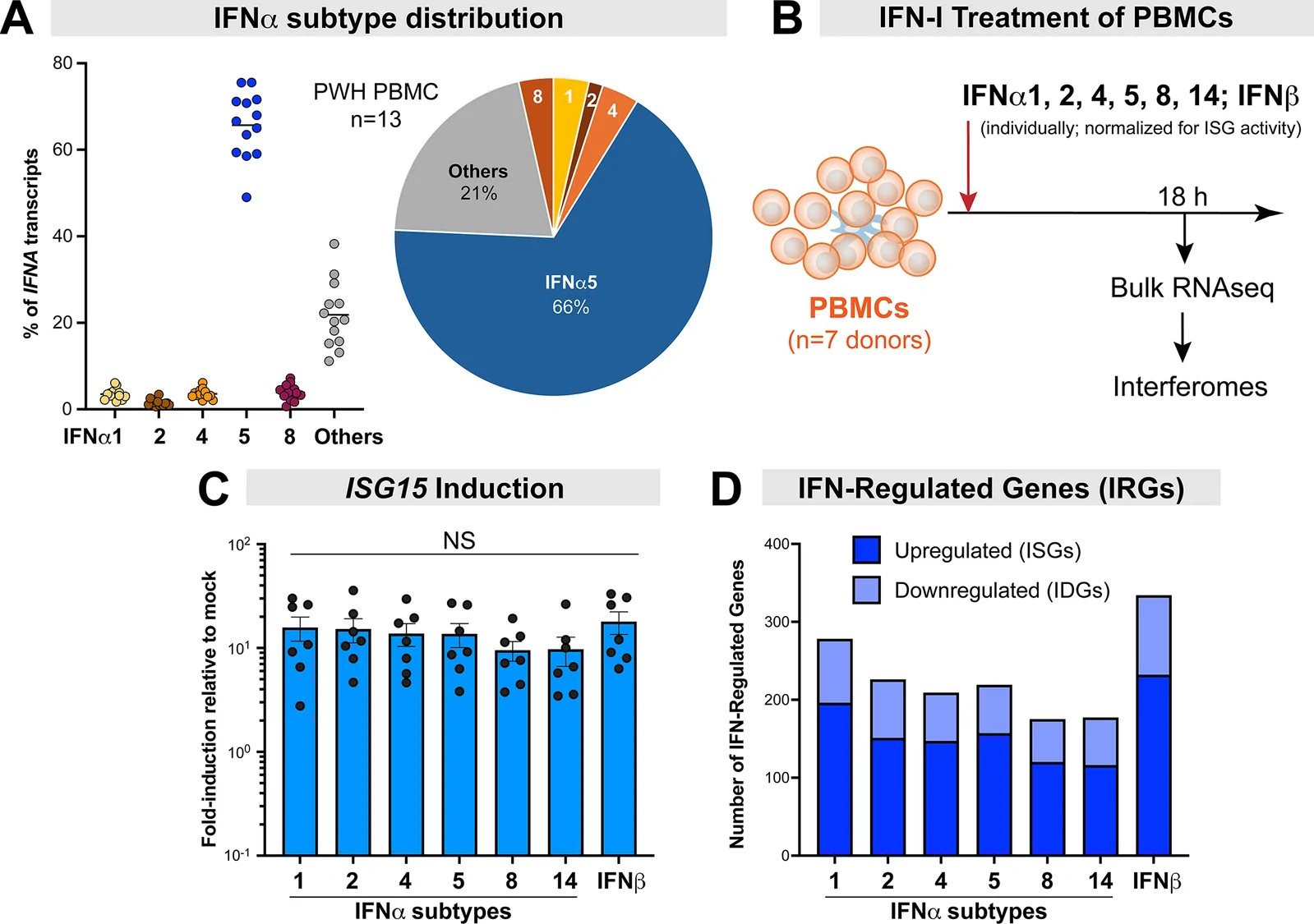
Genes altered by diverse IFN-Is in PBMCs *in vitro*. (**A**) Proportion of *IFN* transcripts in PBMCs of 13 PWH as described in our previous study. The five IFNα subtypes chosen were significantly induced in PBMCs of PWH vs matched controls. (**B**) IFN-I treatment of PBMCs. IFN-Is were normalized for *ISG15*-ISRE activity prior to treatment of PBMCs (*n* = 7 donors). PBMC RNA was isolated after 18 h of culture and subjected to bulk RNA-seq to determine the corresponding interferomes. (**C**) *ISG15* induction relative to mock-treated cultures. Each dot corresponds to a PBMC donor. Differences were evaluated by the Kruskal-Wallis test, followed by Dunn’s multiple comparisons test. NS, not significant. (**D**) Enumeration of IFN-regulated genes (IRGs), subdivided into those upregulated (ISGs) or downregulated by the IFN-I tested. IRGs were determined based on a 1.5-fold cut-off relative to mock, and FDR < 0.2.

The IFNα subtype genes that were significantly upregulated in PWH from our previous study*—IFNA1*, *IFNA2*, *IFNA4*, *IFNA5,* and *IFNA8*—accounted for 78% of *IFNΑ* transcripts among PWH (18). *IFNΑ5* was the most prominent, accounting for two-thirds of *IFNΑ* transcripts (Fig. 1A). The interferomes associated with these IFNα subtypes in peripheral blood remain unknown. To address this knowledge gap, recombinant IFNs were normalized in an *ISG15*-ISRE reporter cell line, then used to stimulate PBMCs *in vitro*. This normalization procedure is similar to our prior study, requiring more protein concentration for IFNα subtypes with lower signaling capacity such as IFNα1 (12, 21). The PBMCs were obtained from seven donors (mean age 42.7, 43% female; Table S1). At 18 h post-stimulation, PBMC RNA was extracted and subjected to RNA-seq (Fig. 1B). As controls, we included IFNβ, which was not detected in the blood of PWH, and IFNα14, a potent IFNα subtype also not upregulated. After filtering and normalization (Fig. S1A), we obtained an average of 12.6 million sequence reads per donor and condition (Table S1). Differentially regulated genes (DEGs), identified using an FDR cut-off of 20% and absolute fold-change >1.5, clustered in a donor-dependent manner by principal component analysis (Fig. S1B).

As expected from how the IFNs were titrated and normalized, IFNα subtypes and IFNβ induced *ISG15* to similar extents (mean 13.6-fold; ANOVA, *P* > 0.05; Fig. 1C). However, the overall number of IFN-regulated genes (IRGs, which define genes that were either up- or downregulated) varied (Fig. 1D; Table S2). While a mean of 214 genes was altered for the individual IFNα subtypes tested, IFNβ altered 56% more genes (Fig. 1D). On average, 69% of IRGs were upregulated and thus considered as interferon-stimulated genes (ISGs). We will refer to the remaining fraction, the genes downregulated by IFN-Is, as IDGs (IFN-I downregulated genes); other studies also refer to these as IFN-repressed genes (IRepG) (22).

**Fig 2:**
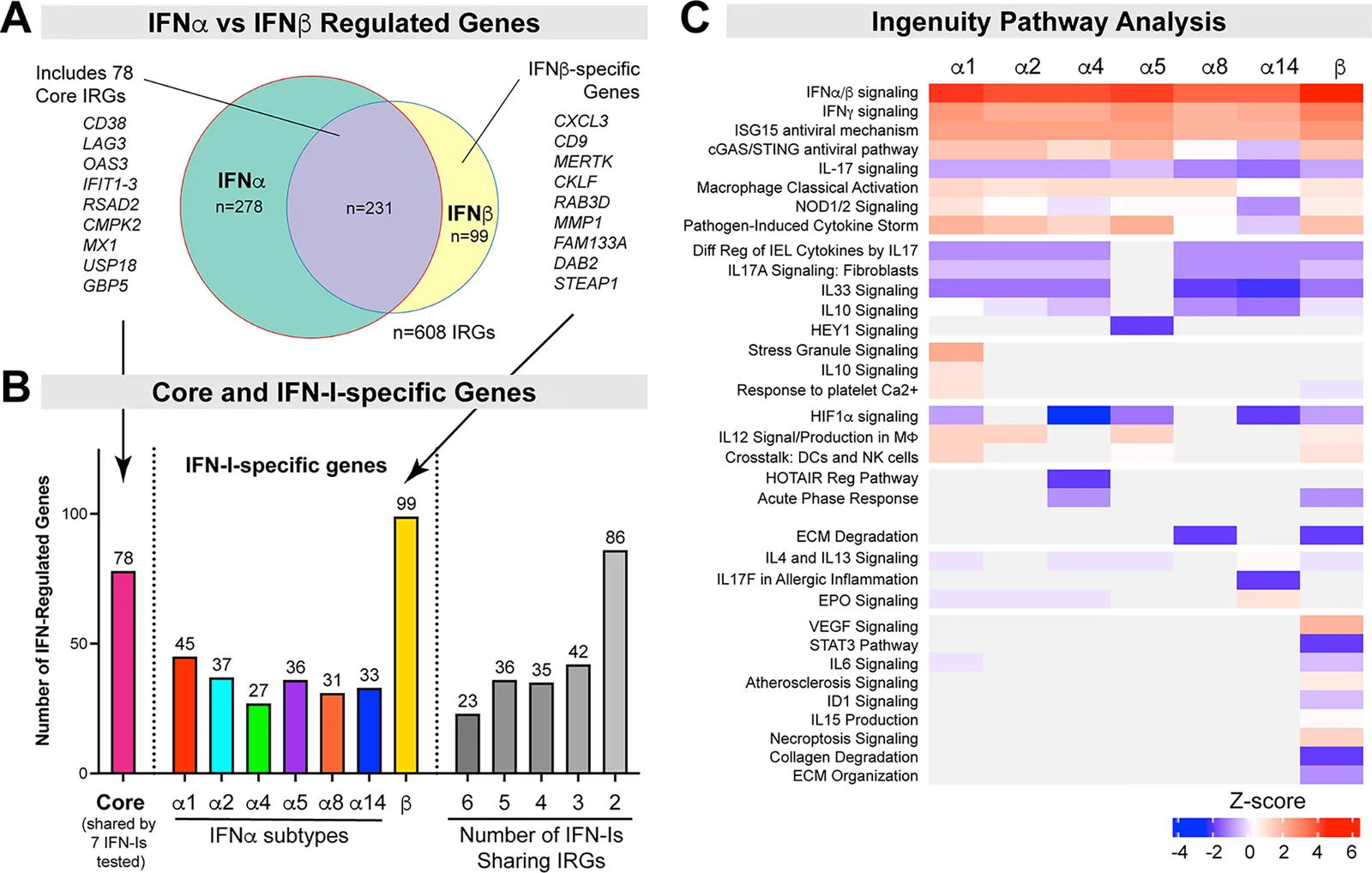
Identification of Core and IFN-I-specific IRGs. (**A**) Venn diagram showing the overlap between IRGs induced by IFNβ vs combined IFNα subtypes. (**B**) Core and IFN-I-specific genes were identified based on the IRG list. Core IRGs (*n* = 78) correspond to genes consistently regulated by all the IFN-Is tested. IRGs induced by two to six IFN-Is are shown in gray and parsed out in more detail in Fig. S2. (**C**) Ingenuity Pathway Analysis based on IRGs for each IFN-I tested. *Z* values were plotted as a gradient to display the level of activation (red) or inhibition (blue).

Despite similar induction of the canonical *ISG15* (Fig. 1C), the genes altered by IFNα subtypes and IFNβ only partially overlapped. About 30% of the genes altered by IFNβ were not significantly altered by any of the six IFNα subtypes tested (Fig. 2A). Within the IFNα subtypes, only 78 (37% of the interferomes on average) were significantly altered by each subtype. These genes were designated as “Core IRGs” (Fig. 2B; Table S3). A majority (*n* = 62; 79.5%) of the Core IRGs were canonical ISGs that include well-described antiviral genes, such as *MX1*, *IFIT1*, and *RSAD2* (viperin). The downregulated Core IDGs included chemokines such as *CXCL5* and *CCL1*, as well as diverse immune mediators (*CLEC5A*, *CD93*, and *VSIG4*). Notably, several IRGs (from 27 for IFNα4 to 45 for IFNα1) were significantly altered by only one IFNα subtype (Fig. 2B; Table S4), while some IRGs were regulated by 2 to 6 out of 7 IFNα subtypes tested (Fig. S2). These data indicated qualitative differences in IRG induction between the IFN-Is tested.

To test if the alteration of distinct genes by IFN-I may trigger diverse pathways, we subjected the individual IFN-I IRGs to Ingenuity Pathway Analysis (Fig. 2C; Table S5) (23). As expected, all IFN-Is were associated with activation of “interferon signaling” and signaling via cGAS-STING. While all IFN-Is were linked to downregulation of IL17 signaling, IFNα5 appeared to be less associated with other IL17-associated pathways, as well as IL33 and IL10 signaling. Multiple pathways were associated only with IFNβ, and certain IFNα subtypes were uniquely associated with “stress granule signaling” (IFNα1), HEY1 signaling (IFNα5), acute phase response (IFNα4), and degradation of the extracellular matrix (IFNα8).

### Core ISGs linked to T-cell activation, immune exhaustion, and metabolic dysfunction are associated with HIV-1 pathogenesis

**Fig 3:**
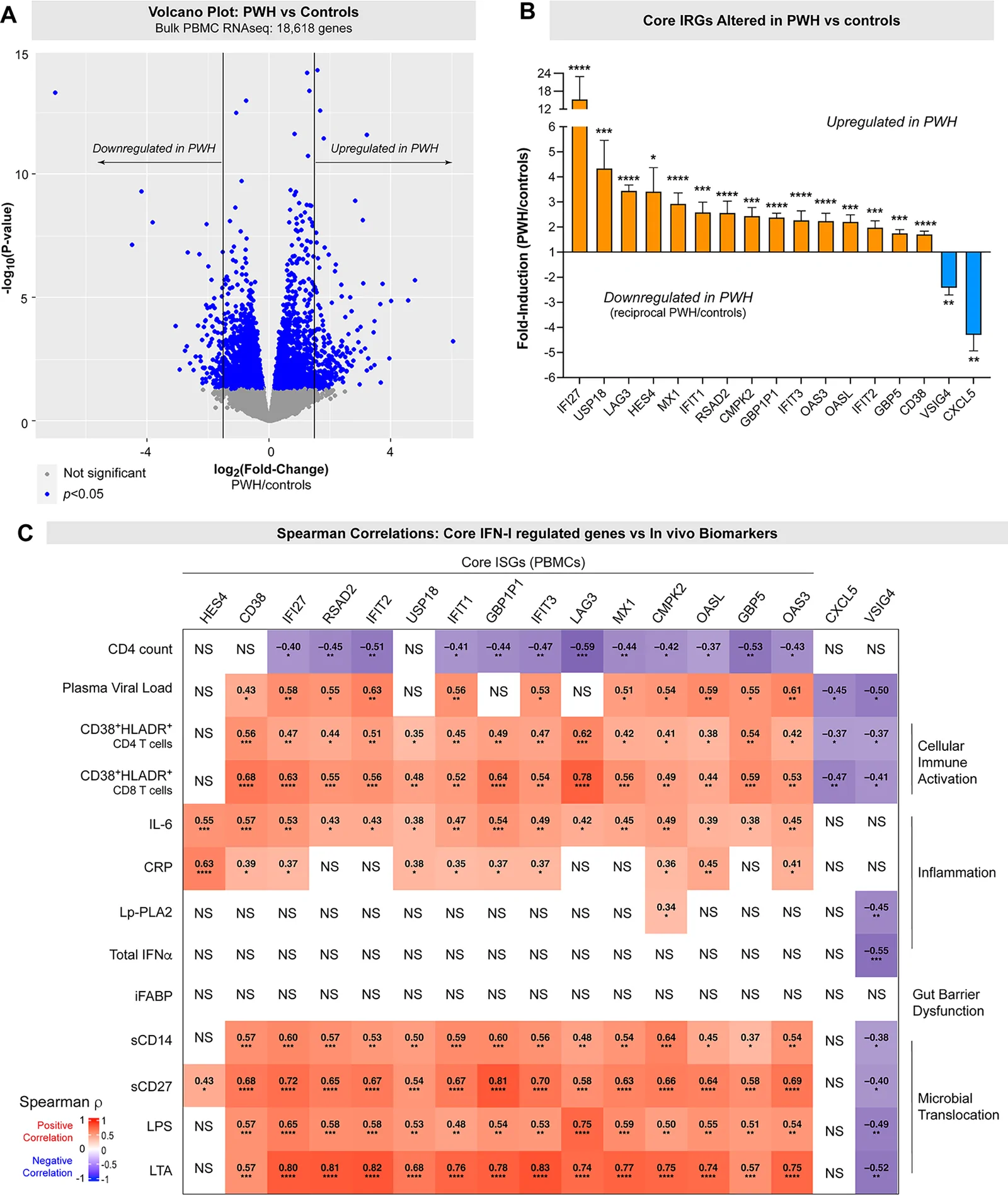
Core IRGs linked to HIV-1 pathogenesis. (**A**) Volcano plot of PBMC differentially regulated genes (DEGs) in a clinical study of 21 PWH and 13 matched controls. DEGs were defined based on a 1.5-fold change and FDR < 0.05. (**B**) Core IRGs (from Fig. 1B) altered in PWH vs controls. TMM-normalized values were compared using two-tailed Student’s *t*-test. (**C**) Correlogram of the 17 Core IRGs (15 ISGs and 2 IDGs) against biomarkers of cellular activation, inflammation, gut barrier dysfunction, and microbial translocation. Gradients approximate the extent of positive (red) or negative (purple) correlation based on Spearman’s rho values. For panels B and C, *, *P* < 0.05; **, *P* < 0.01; ***, *P* < 0.001; and ****, *P* < 0.0001.

We next tested if core and IFNα-subtype-specific IRGs identified *ex vivo* (Fig. 2B) were altered during untreated chronic HIV-1 infection *in vivo*. For this purpose, we utilized samples and published data from our previously completed clinical study involving PWH who were not on ART and age/sex-matched controls (12, 18, 24–26). RNA was isolated from bulk PBMCs from 21 PWH and 13 controls for RNA-seq (Table 1). Following TMM normalization and filtering (27, 28), 18,618 genes were analyzed from an average of 43 million reads/individual. Of 777 (4.2%) genes that were differentially regulated (FDR < 5%) in PWH vs controls, 125 (0.67%) were altered by more than 1.5-fold (Fig. 3A).

**TABLE 1:** Systemic inflammation biomarkers in a clinical cohort of PWH and age/sex-matched controls^*a*^

	CD4 count (cells/mL)	Sex	Age (yr)	Plasma VL (copies/mL)	CD38^+^ HLA-DR^+^ CD4 T cells (%)	CD38^+^ HLA-DR^+^ CD8 T cells (%)	IL-6 (pg/mL)	CRP (mg/mL)	Lp-PLA2 (ng/mL)	IFNα total	iFABP (pg/mL)	sCD14 (ng/mL)	sCD27 (U/mL)	LPS (pg/mL)	LTA (OD)	RNA-seq counts
C119	760	M	27	N/A	2.03	2.6	1.01	1.53	216.31	2.74	1,334.07	1,856.05	29.66	12.54	0.06	45,327,495
C138	728	M	29	N/A	1.18	2.32	2.16	0.25	236.77	2.35	1,862.75	1,633.46	36.19	9.52	0.18	42,494,229
C178	736	M	33	N/A	0.83	1.59	0.51	0.65	156.18	0.54	2,363.33	2,073.05	43.24	9.8	0.38	39,229,405
C255	588	M	34	N/A	0.9	1.61	0.69	0.7	217.55	0.95	1,516.51	1,605.46	29.35	14.48	0.1	48,396,743
C278	532	M	23	N/A	0.76	1.77	0.73	0.44	149.06	0.96	2,800.08	2,145.85	32.47	7.45	0.12	43,077,809
C404	1,071	F	29	N/A	0.51	1.75	1.35	12.37	ND	1.11	547.44	1,184.07	37.42	9.7	0.19	37,685,224
C493	672	F	28	N/A	0.58	0.42	0.19	0.79	47.7	1.03	1,742.71	1,767.86	31.54	5.99	0.14	36,312,036
C582	976	M	54	N/A	ND	ND	0.7	0.23	77.15	1.19	4,061.74	1,756.66	24.95	8.13	0.07	38,150,597
C708	468	M	47	N/A	1.62	4.62	0.27	0.37	106.9	2.32	2,436.11	1,336.66	29.35	14.11	0.12	49,087,363
C716	1,035	M	27	N/A	0.87	1.07	0.47	0.75	36.85	1.31	1,024.76	1,492.06	23.36	11.78	0.16	43,273,431
C914	690	F	43	N/A	2.61	1.74	1.21	2.73	55.14	0.99	3,083.56	1,679.66	39.27	8.78	0.17	44,078,238
C947	480	M	25	N/A	1.8	3.68	0.78	0.61	128.6	0.39	2,340.34	1,788.86	29.97	7.66	0.1	43,126,507
C972	1,035	F	51	N/A	0.67	3.61	0.48	0.61	136.66	1.03	4,100.05	1,814.06	31.54	11.93	0.13	61,066,960
H124	400	M	48	8,400	2.41	19.78	4	2.88	163.62	1.1	2,373.55	2,239.65	36.81	17.92	0.32	34,882,119
H132	532	M	25	26,000	1.85	22.67	1.39	4.21	170.75	1.32	4,919.88	1,784.66	74.29	19.87	0.2	42,880,928
H154	400	F	58	22,000	1.74	13.54	5.09	11.32	43.05	1.78	2,718.34	1,917.65	51.7	14.49	0.31	50,049,554
H188	836	M	44	133,000	4.47	11.88	1.22	1.92	204.85	1.37	1,224	ND	52.9	ND	ND	38,802,586
H196	621	M	29	81,500	2.93	15.37	1.73	2.9	257.54	1.92	2,240.74	2,274.65	51.7	18.85	0.21	56,664,750
H217	744	M	22	25,200	0.62	5.47	0.56	0.2	205.47	3.03	3,634.9	ND	35.88	12.63	ND	38,231,176
H286	693	F	52	3,850	1.76	17.12	1.19	2.54	98.84	4.44	6,005.33	1,889.65	43.24	14.26	0.22	34,642,327
H307	624	M	34	9,180	10.35	38.33	1.16	0.97	91.71	1.83	2,116.29	1,506.06	56.77	15.06	0.23	46,114,053
H323	429	M	54	9,440	1.13	4.81	1.02	1.28	184.08	2.84	2,378.27	1,791.66	33.71	11.25	0.17	29,293,992
H391	238	F	29	196,000	3.73	20.84	1.52	0.68	149.98	1.1	2,424.63	3,317.63	62.96	27.02	0.64	40,630,821
H428	460	M	28	25,100	5.45	29.88	1.78	1.37	204.54	2.08	1,686.52	2,102.45	36.19	20.29	0.28	45,260,809
H456	1,248	M	46	7,400	1.21	6.49	2.36	2.36	128.91	0.97	2,938.7	ND	42.63	9.67	ND	44,621,033
H594	338	M	46	88,600	6.61	14.81	1.55	2.8	321.39	1.29	3,677.08	2,074.45	106.17	ND	0.43	43,618,949
H622	340	M	33	112,000	4.63	19.73	1.96	2.21	143.48	1.69	2,890.2	2,001.65	52.3	16.85	0.27	38,895,573
H648	420	M	31	2,880	1.88	5.22	0.52	0.21	114.03	2.9	3,155.07	1,450.06	29.35	11.84	0.31	37,769,057
H683	504	M	25	59,500	2.76	9.18	0.43	0.8	255.68	2.21	2,411.01	1,457.06	38.04	19.34	0.29	35,837,517
H819	527	M	27	4,670	4.53	20.88	0.98	0.32	72.5	1.52	2,856.36	2,085.65	43.24	15.9	0.4	34,991,429
H825	364	M	34	43,200	2.22	5.65	0.6	1.25	230.26	3.13	3,057.21	1,927.45	31.22	16.48	0.2	45,683,221
H839	250	F	39	64,900	5.29	14.34	3.98	4	289.77	1.81	5,775.47	2,948.03	62.96	19.04	0.26	76,508,564
H965	221	F	26	155,000	6.6	38.52	1.43	0.87	101.01	1.7	529.5	2,508.44	50.8	16.7	0.34	35,972,629
H998	782	F	25	119,000	1.39	16.72	0.87	0.46	152.15	4.68	1,574.88	1,969.45	49.6	8.29	0.26	37,948,893

^*a*^ N/A, not applicable; ND, no data available.

Ingenuity Pathway Analysis showed increased IFN responses among PWH (*Z*-score = 2.0, FDR < 5%). This provided the rationale to further investigate the specific IRGs we detected in PBMCs after IFN-I treatment *in vitro* (Fig. 2B). Sixteen out of 62 (26%) Core ISGs were significantly upregulated in PBMCs of PWH (Fig. 3B; Table S3). Many of these core ISGs had known antiviral activity: *MX1*, *RSAD2*, *GBP5*, *OAS*, and *IFIT1-3* (29). One gene, *PRAME*, was excluded from subsequent analyses as all control samples and a substantial fraction of PWH (14/21; 67%) did not express the gene. Notably, 2 out of 16 Core IDGs from *in vitro* stimulation, *CXCL5* and *VSIG4*, were also significantly downregulated *in vivo* among PWH relative to controls (Fig. 3B; Table S3).

We next determined if the 17 Core IRGs (15 ISGs and 2 IDGs) correlated with CD4 T-cell count, plasma viral load, systemic immune activation (%CD38^+^HLA-DR^+^ CD4 and CD8 T cells), inflammation (IL6, CRP, and sCD27), epithelial barrier dysfunction (iFABP), microbial translocation (sCD14, LPS, and LTA), and total plasma IFNα levels that were measured in prior studies (12, 24, 30, 31) (raw data sets are provided in Table S6). The heatmap in Fig. 3C highlights the Spearman correlation coefficient for these pairwise analyses. As we previously reported for *APOBEC3G*, *Tetherin/BST-2*, and *MX2* (18), multiple antiviral ISGs positively correlated with plasma viral load (among PWH) and inversely with CD4 T-cell count (entire cohort). These ISGs did not correlate with total IFNα levels (Fig. 3C). Core ISGs, including those involved in T-cell activation (*CD38*) (32, 33), immune exhaustion (*LAG3*) (34, 35), and dNTP metabolism (*CMPK2*) (36), significantly correlated with inflammation (IL-6) and T-cell activation. By contrast, Core IDGs *CXCL5* and *VSIG4* negatively correlated with plasma viral load and cellular immune activation (Fig. 3C). Core ISGs also positively correlated with microbial translocation biomarkers, but *VSIG4* (but not *CXCL5*) showed a negative correlation. *VSIG4* expression also negatively correlated with IFNα levels. None of the IRGs showed a correlation with iFABP.

### *MUC1*, an IFNα5-regulated gene, is associated with T-cell exhaustion and systemic inflammation

**Fig 4:**
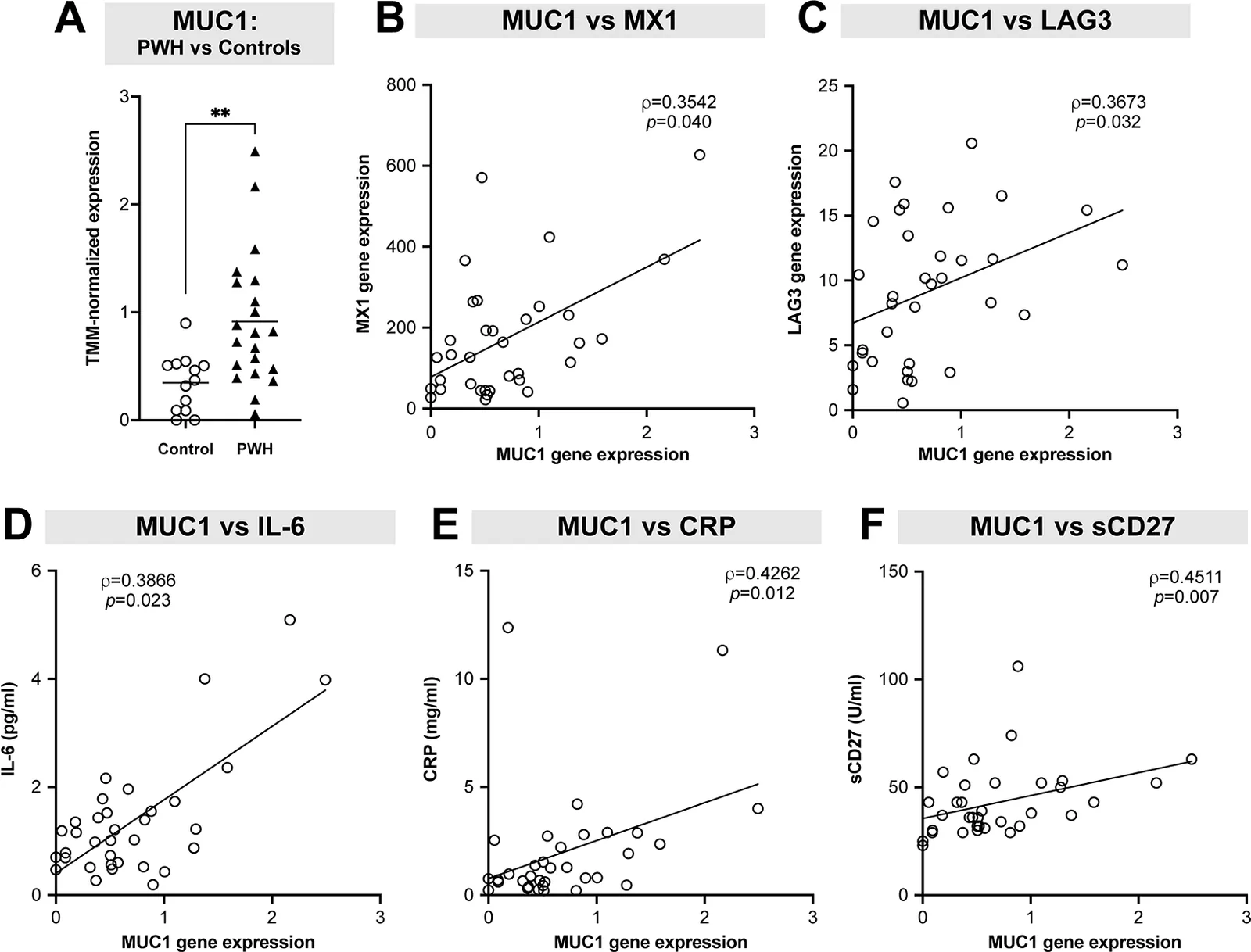
*MUC1* and systemic inflammation in PWH. (**A**) *MUC1* transcript levels in PBMCs of PWH (*n* = 21) vs age/sex-matched controls (*n* = 13). Lines correspond to mean TMM-normalized values. Groups were compared using the two-tailed Mann-Whitney test. **, *P* < 0.01. Correlations were tested between *MUC1* transcript levels and (**B**) *MX1*, a canonical ISG; (**C**) *LAG3*, a marker of T-cell exhaustion; and plasma inflammation markers (**D**) IL-6; (**E**) C-reactive protein; and (**F**) soluble CD27. For panels B–F, best-fit lines were added, and relationships were evaluated using Spearman’s rank correlation analysis.

A handful of IFNα subtype-specific genes that included *SULT1B1*, *SNAI1*, *MUC1*, and *VIT* were significantly altered in PWH relative to controls *in vivo* (Table 2). Among these genes, only *MUC1* (Fig. 4A) was previously linked to HIV-1 infection, where the gene product, Mucin-1, blocked DC-SIGN-mediated transmission of HIV-1 from dendritic cells to CD4 T cells (37). *MUC1* was most strongly induced by IFNα5, with the highest *P* value and FDR, but non-adjusted analyses and lower statistical thresholds suggest that it may also be regulated by other IFN-Is, particularly IFNβ (Fig. S3). Consistent with its ISG status, *MUC1* peripheral blood transcript levels strongly correlated with canonical ISGs such as *MX1* (Fig. 4B) *in vivo*.

**TABLE 2:** IFNα subtype-specific genes significantly altered in PWH PBMCs^*a*^

Gene	Complete gene name	*In vitro*: PBMCs				*In vivo*: PWH vs controls		
		IFNα subtype	Log_2_FC	*P* value	FDR	Log_2_FC	*P* value	FDR
SULT1B1	Sulfotransferase family 1B member 1	IFNα1	−2.213	0	0.002	−0.763	0	0.009
RPS24P8	Ribosomal protein S24 pseudogene 8	IFNα2	−2.243	0.009	0.079	−1.532	0	0.013
BX679664.3		IFNα4	−2.412	0.012	0.103	−2.122	0	0.009
SNAI1	Snail family transcriptional repressor 1	IFNα5	3.341	0.001	0.015	−1.911	0.001	0.025
MUC1	Mucin 1, cell surface associated	IFNα5	2.255	0	0	1.491	0.001	0.027
VIT	Vitrin	IFNα8	−2.504	0.012	0.118	1.767	0	0.008
G0S2	G0/G1 switch 2	IFNα14^*a*^	−2.172	0.008	0.078	−2.078	0	0.006
CXCL8	C-X-C motif chemokine ligand 8	IFNα14^*a*^	−2.167	0	0.001	−1.281	0.005	0.088
HMGB1P10	High mobility group box 1 pseudogene 10	IFNα14^*a*^	−2.89	0.028	0.178	−1.421	0	0

^*a*^ IFNα14 was not significantly upregulated in PWH vs controls in our prior study (18).

Mucin-1 is a transmembrane protein heavily decorated with O-linked glycans (38). While typically expressed on mucosal epithelial cells where it acts as a protective, lubricating barrier to defend against pathogens and dehydration, Mucin-1 was also detected in lymphocytes, where it may play a role in oncogenesis by promoting T-cell exhaustion (39). In the PWH study cohort (Table 1), *MUC1* levels correlated with a canonical T-cell exhaustion marker, *LAG3* (Fig. 4C). We also detected a significant positive correlation with IL-6, CRP, and sCD27 (Fig. 4D through F). We did not detect significant correlations with the other biomarkers in Fig. 3C, including T-cell activation and microbial translocation markers LPS and LTA (*P* > 0.05; data not shown). Altogether, these data link *MUC1* to IFN-mediated systemic inflammation and T-cell exhaustion in chronic HIV.

### IFNα5 upregulates Mucin-1 specifically in peripheral blood T cells

**Fig 5:**
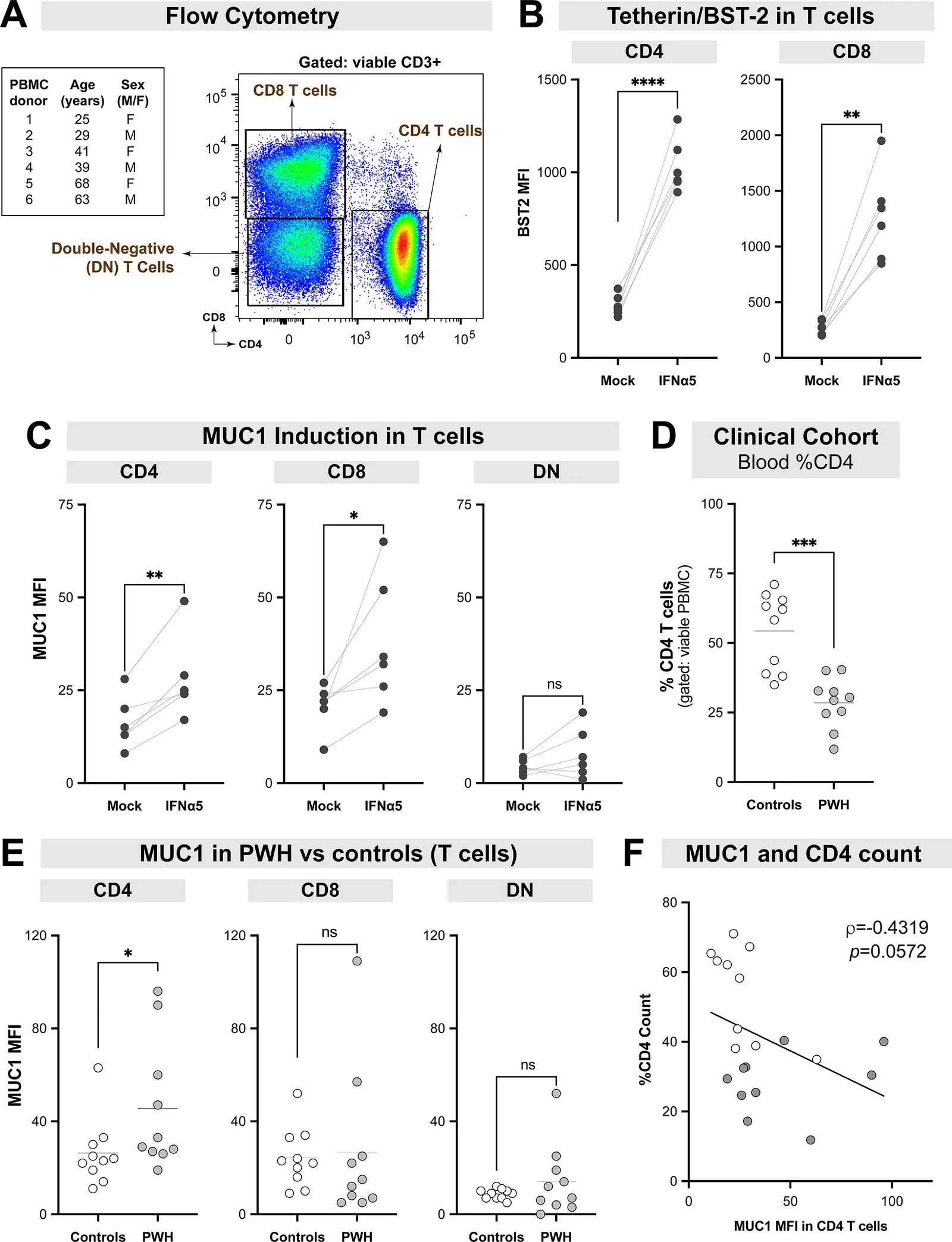
Mucin-1 expression in immune cells. (**A**) PBMCs from six donors were treated *in vitro* with IFNα5 (3.62 nM) and subjected to flow cytometry after 2 days. The gating strategy for CD3^+^ T cells was shown. (**B**) As a positive control, a canonical ISG, Tetherin/BST-2 was evaluated. (**C**) Mucin-1 expression was evaluated by flow cytometry in various PBMC CD3^+^ T cells. Mean fluorescence intensity (MFI) values are shown for each of seven donors (black dots). DN = double-negative for CD4 and CD8. Pairwise comparisons were performed using a two-tailed paired Student’s *t*-test. *, *P* < 0.05; **, *P* < 0.01, ****, *P* < 0.0001; and ns, not significant. (**D**) Frequency of CD4 T cells in PBMCs of PWH vs controls (see Table 3 for more details). ***, *P* < 0.001 by the 2-tailed unpaired Mann-Whitney test. (**E**) Mucin-1 cell surface density in CD4, CD8, and DN CD3^+^ T cells of PWH vs controls. Comparisons were performed using the two-tailed unpaired Mann-Whitney test. (**F**) Correlation between Mucin-1 MFI in CD4 T cells and % CD4 T cell frequency from panel B. Spearman’s rho values are shown.

We next determined if IFNα5-mediated induction of Mucin-1 occurs at the protein level, and if so, which PBMC cell types were involved. PBMCs (*n* = 6 donors without HIV) were treated with 3.62 nM IFNα5, the same concentration used for the PBMC interferome studies (Fig. 1B), then subjected to flow cytometry (Fig. 5A). As a positive control, we also evaluated a canonical ISG, Tetherin/BST-2 (40). As expected, Tetherin/BST-2 was significantly upregulated by IFNα5 in immune cells including T cells (Fig. 5B) and non-T cells (*P* < 0.01, data not shown). Mucin-1 surface expression was significantly induced by IFNα5 on CD4^+^ and CD8^+^ T cells (Fig. 5C), but it was not detected on CD19^+^ B cells, CD14^+^ monocytes or on immune cell subsets enriched for DCs (CD3^−^CD19^−^CD14^−^HLA-DR^+^) or NK/ILCs (CD3^−^CD19^−^CD14^−^HLA-DR^−^) (MFI < 1, data not shown). These data suggested that mainly T cells contributed to the *MUC1* transcript upregulation in PWH PBMCs vs controls (Fig. 4A).

**TABLE 3:** PBMC donor profiles for Mucin-1 immunophenotyping

	Age	Sex	% CD4 T cells	HIV-1 viral load
Controls				
Donor1	49	M	34.96	N/A
Donor2	45	F	65.39	N/A
Donor3	48	F	71.01	N/A
Donor4	24	M	62.12	N/A
Donor5	38	M	43.71	N/A
Donor6	30	F	38.08	N/A
Donor7	30	F	67.27	N/A
Donor8	30	F	58.27	N/A
Donor9	52	M	63.22	N/A
Donor10	37	M	38.89	N/A
Mean	38.3		54.3	
Viremic				
Donor1	49	M	17.23	76,100
Donor2	48	F	40.38	19,900
Donor3	39	F	30.44	64,900
Donor4	29	M	24.64	70,300
Donor5	33	M	40.08	187,000
Donor6	35	F	25.44	17,700
Donor7	32	F	29.37	27,900
Donor8	35	F	32.76	17,100
Donor9	51	M	11.83	34,400
Donor10	32	M	32.44	85,400
Mean	38.3		28.5	60,070

### Mucin-1 is upregulated in peripheral blood CD4^+^ T cells of untreated, chronically infected PWH

To confirm that Mucin-1 was upregulated in peripheral blood T cells of PWH, we obtained archived PBMCs from viremic PWH (mean viral load = 60,070 copies/mL) and controls (*n* = 10 per cohort; Table 3). The two cohorts were matched for age (mean age = 38.3; *P* > 0.05), and sex (50% female), and the frequencies of CD4^+^ T cells were significantly lower among PWH (Fig. 5D). The proportions of Mucin-1^+^ cells were not significantly different in CD4^+^ and CD8^+^ T cells between PWH and controls (*P* > 0.05). However, Mucin-1 MFI levels in CD4^+^ T cells, but not in CD8^+^ T cells, were significantly higher in PWH (Fig. 5E), and we detected a trend (*P* = 0.052) for lower blood CD4 T-cell frequencies in individuals with higher Mucin-1 CD4 T-cell MFI levels (Fig. 5F).

## DISCUSSION

Type I interferons (IFN-Is) are essential for early antiviral defense, but persistent IFN-I signaling has been linked to pathogenic outcomes (5, 12, 41). Our understanding of how IFN-Is trigger disease remains incomplete. The extensive diversity of IFNα subtypes encoded in the human genome adds complexity to tackling this issue. Although all IFNα subtypes bind to the same receptor IFNΑR, they do so with varying binding affinities, resulting in quantitative differences (9, 21). Normalizing for quantitative differences, IFNα subtypes could still trigger distinct gene programs in isolated cell populations, suggesting qualitative differences (12). Here, we tested if qualitative differences would still be observed in mixed cell populations such as PBMCs, which account for the majority of interferomics studies. Our data revealed both shared (core) and unique genes altered by multiple IFN-Is, suggesting common properties and qualitative differences between them.

We utilized chronic HIV infection as a platform to study how shared and unique genes regulated by IFNα subtypes may impact pathogenic outcomes *in vivo*. In the systemic circulation, persons with HIV (PWH) upregulated multiple IFNα subtypes, but not IFNβ (18, 20). We identified multiple core ISGs that were significantly upregulated in peripheral blood of PWH compared to age/sex-matched controls. The majority of these core ISGs were linked to the antiviral response, but three were associated with cellular immune functions. CD38 and LAG3 are classic markers of T-cell activation and exhaustion, respectively, that were previously reported to be elevated among PWH (32–34, 42). Consistent with this, blockade of IFNΑR signaling in humanized mice persistently infected with HIV-1 reversed the T exhaustion phenotype and restored immune function (43, 44). *CMPK2* encodes for a mitochondrial enzyme that facilitates the conversion of dUMP to dUTP (36), and has been reported to exhibit antiviral properties (45). Notably, increased CMPK2 expression has also been linked to cellular senescence (36, 46). IFNs have been linked to the natural aging process (47), whereas chronic HIV promotes accelerated aging (48, 49). It remains to be determined if CMPK2 contributes to accelerated aging among PWH. Altogether, these data suggest that IFNs may drive pathogenic outcomes among PWH by augmenting T-cell activation, exhaustion, and metabolic dysfunction.

We observed two IFN-downregulated genes, *CXCL5* and *VSIG4*, that were significantly inhibited in PWH PBMC. In addition to their classical roles as gene inducers, IFN-I are known to also repress host genes such as hnRNPA0 via JAK1/2-dependent pathways (50), but the molecular mechanisms remain unclear. Notably, *CXCL5* and *VSIG4* expression correlated negatively with plasma viral load, suggesting a protective effect. Notably, these two genes are predominantly expressed by macrophages (51–53). We hypothesize that CXCL5, a neutrophil chemoattractant (51), may coordinate localized innate immune responses without broadly amplifying systemic inflammation or tissue injury (54). VSIG4^+^ tissue-resident macrophages can clear complement-tagged pathogens and inflammatory debris, potentially lowering the systemic stimulus load (52, 53). Consistent with this, *VSIG4* negatively correlated with plasma microbial translocation biomarkers LPS, LTA, sCD14, and sCD27. As very limited information currently exists on how these genes influence HIV-1 infection, mechanistic studies would be an important focus of future studies.

Our *in vitro* transcriptomic data clearly show interferome differences between the IFNα subtypes, strengthening the evidence for qualitative differences. However, the magnitude of differences in PBMCs was more subdued compared to purified gut CD4 T cells and alveolar epithelial cells (12, 13). For example, IFNα14 upregulated substantially more genes than other IFNα subtypes in gut CD4 T cells (12); but this was not observed in PBMCs. We speculate that having a mixed cell population may have masked cell type-specific differences. Surprisingly, in PBMCs, IFNα1 altered the most genes among the IFNα subtypes tested. IFNα1, which technically corresponds to identical proteins produced by *IFNΑ1* and *IFNΑ13*, has defective binding to IFNΑR2 (55). Normalizing for protein levels, it should alter the least number of genes *in vitro*. Here, we utilized eightfold higher amounts of IFNα1 (nM) to normalize its signaling capacity to other IFNα subtypes. This suggested that if expressed at high levels, IFNα1 may direct distinct biological programs. For example, pathway analyses suggested that it may uniquely impact IL10 signaling. We also note that the interferome data obtained were only from a single time point (18 h post-stimulation) to be consistent with our prior report on purified CD4 T cells (12). Interestingly, several waves of ISG induction were reported with IFNβ in T cells (56). In primary intestinal epithelial cells, IFN-I vs IFN-III showed distinct kinetics of ISG induction (57). Understanding the kinetics of ISG induction or IDG repression between the IFNα subtypes will be an important focus of future studies.

The functional consequences of diverse IFNα subtypes are immediately apparent in murine studies, where treatment of Friend retrovirus-infected mice with distinct IFNα subtypes showed differential effects on NK cell and CD4+ T-cell function (58, 59). However, it remains unknown if individual human IFNα subtypes could direct unique gene programs linked to protection or pathogenesis *in vivo*. In PBMCs from PWH, IFNα5 accounted for the majority of *IFNΑ* transcripts, implying that it is the dominant IFNα subtype driving chronic IFN signaling in the systemic circulation. IFNα5 is one of the least potent IFNα subtypes against HIV-1 (21), suggesting that it may be a mechanism for diminished innate immune responses among PWH.

Our PBMC data contrasts from plasmacytoid DCs stimulated with HIV-1 virions, which showed upregulation of *IFNA1/13*, *5*, *2*, *14*, and *8* on the centromeric half of the *IFNA* locus, suggesting an epigenetic component (21). Thus, non-pDC cell types in PBMCs may be driving the *IFNA5* bias. It remains to be determined how IFNα subtypes regulate the transcriptome of individual immune cell types in PBMC. Of note, the current *IFNA* subtype distribution is based on transcriptional data. Due to the high sequence similarity between IFNα subtypes (mean pairwise difference of 16.6%), assay platforms that could robustly distinguish individual IFNα subtype proteins through antibodies (PBL Assay Science, personal communication) or aptamers (60) in plasma remain unavailable. IFNα-subtype-specific protein assays will also need to account for the very low levels of IFNα in plasma of PWH, estimated at ~4.27 pg/mL (19). While IFNα protein levels do correlate with *IFNA* transcripts (19, 61), the extent that this applies to individual IFNα subtypes remains unknown. Nevertheless, our transcriptomic profiling of IFNα-treated PBMCs *in vitro* revealed that IFNα5 more significantly induced *MUC1*, which in turn correlated with clinical inflammation outcomes (see below). The encoded protein, Mucin-1, may have antiviral properties, particularly against influenza A and SARS-CoV-2 (62). In fact, IFNα5 was superior to most IFNα subtypes in inhibiting these viruses in alveolar epithelial cells (63, 64). It would be of interest to determine if Mucin-1 would explain why IFNα5 had higher potency against respiratory viruses.

In our clinical cohort, *MUC1* expression correlated with the systemic inflammatory mediator IL-6. Through STAT3-dependent signaling, IL-6 can drive CRP production in hepatocytes and promote T-cell exhaustion (65, 66). Follow-up immunophenotyping revealed that Mucin-1’s biological impact on HIV is linked to higher expression in circulating CD4^+^ T cells on a per-cell basis. *MUC1* correlated strongly with *LAG3* in our clinical cohort, and there is evidence in murine cancer models that Mucin-1 promotes T-cell exhaustion (39). Studies are now ongoing to determine if Mucin-1 could directly facilitate CD4^+^ T-cell exhaustion during chronic HIV. Of note, altered O-glycan variants of Mucin-1 in various tumors are considered an important therapeutic target (67). The cytoplasmic tail of Mucin-1 could activate STAT3 (and vice versa) to promote cell survival and is also a therapeutic target in dozens of oncology clinical trials (68, 69). HIV-1 infection and IFNα therapy have been shown to remodel host immune-cell glycosylation patterns (70, 71). Thus, it would be of interest to investigate the Mucin-1 glycoform(s) present in CD4^+^ T cells of PWH, and whether such Mucin-1 glycoforms could inform viral reservoir targeting strategies.

In conclusion, our findings expand our understanding of the biological properties of diverse IFN-Is, providing novel insights on how IFNα subtypes may facilitate systemic inflammation and pathogenesis during chronic HIV-1 infection. The IFNα subtype-specific PBMC interferomes described here could also be useful in the study of “interferonopathies,” as well as other viral infections for which an IFN-I signature is dominant. Further studies on Mucin-1, as well as CMPK2, CXCL5, and VSIG4, may unravel novel mechanisms to disrupt IFN-mediated pathogenic mechanisms that could potentially restore immune function among PWH.

## MATERIALS AND METHODS

### Study participants

Cryopreserved PBMCs from PWH and age- and sex-matched people without HIV were obtained from study participants enrolled in prior completed IRB-approved clinical studies (Table 1, Colorado Multiple Institutional Review Board [COMIRB] #11-0164; Table 3, COMIRB #98-0083 and #11-1164). All study participants voluntarily gave written, informed consent. Inclusion and exclusion criteria and the collection, storage, and processing of PBMC and plasma have been extensively detailed in previous publications (31, 72–74). Clinical parameters that include blood CD4 T-cell counts, plasma viral load, and systemic biomarkers were reported previously (12, 18, 24, 26, 30, 31, 75).

### IFN-I treatment of PBMCs

For transcriptomic studies, deidentified cryopreserved PBMCs were procured from an established commercial source (ZenBio, Table S1). PBMCs (10^3^ cells/well in a 24-well plate) in RPMI with 10% fetal bovine serum, 1% penicillin-streptomycin, and l-glutamine were treated with 100 pg/mL of IFNα14 (PBL Assay Science) and the corresponding nM amounts for the other IFNα subtypes based on an *ISG15*-derived ISRE-reporter cell line titration (iLite type I IFN Assay, Svar Life Science AB, Sweden) (12). Cells were harvested after 18 h for RNA-seq.

### Transcriptomics

RNA-seq libraries were prepared with 500 ng total RNA using QuantSeq 3′ mRNA-Seq Library Prep Kit (Lexogen, Austria, Cat #016.96). The quality of the RNA-seq library was verified using 2100 BioAnalyzer (Agilent, Santa Clara, CA, USA) and then sequenced on a HiSeq 2500 by the Genomics and Microarray Sequencing Core facility at the University of Colorado Anschutz Medical Campus. RNA-seq data were downloaded as FASTQ files and quality was verified via *FastQC* (Babraham Institute, https://www.bioinformatics.babraham.ac.uk/projects/fastqc). The Illumina sequencing adapters were then removed, and base-quality screening was performed using *cutadapt* (https://cutadapt.readthedocs.io/en/stable). The quality-filtered FASTQ files were mapped to the current human genome assembly GRCh38.p12 using *Hisat2* (Johns Hopkins University, https://ccb.jhu.edu/software/hisat2/index.shtml). The Ensembl ID, gene symbol, gene length, and raw gene expression counts were extracted using *featureCounts* from the *Subread* package (Walter + Eliza Hall Institute of Medical Research, http://subread.sourceforge.net).

### PBMC interferomes and pathway analysis

RNA-seq raw counts were acquired for PBMCs treated with seven IFN-Is (IFNα1, 2, 4, 5, 8, 14, and IFNβ) and mock from seven donors. After filtering genes with fewer than five reads per library, the remaining gene counts were normalized using the trimmed mean of *M* values (TMM) normalization method from *edgeR* (version 3.24.3) in R (version 4.5) (27, 28). Using edgeR differential expression analysis, IFN-regulated genes (IRGs) were defined by a 1.5-fold change (FC) cut-off relative to the mock and a false discovery rate (FDR) ≤ 20%. DEGs were used as input for Ingenuity Pathway Analysis (Qiagen) (23).

### Clinical study interferome analysis

The gene-level raw RNA-seq counts were obtained for 14 HIV-1-uninfected donors and 21 PWH. Genes with average read counts ≤ 5 per library were removed. The remaining gene counts were normalized using TMM normalization from edgeR (version 3.24.3) in R (version 4.5). Two-group differential expression (DE) analysis was performed to test the differences between healthy controls and PWH using normalized gene counts in edgeR with default settings and FDR of 5% to control for multiple testing.

### Flow cytometry staining, acquisition, and analysis

Standard flow cytometry staining protocols to determine expression of Mucin-1 and Tetherin/BST-2 on various immune cell subsets at baseline or following *in vitro* culture were followed, as previously detailed (24, 31). Expression was determined on viable cells (Zombie Aqua Live/Dead Fixable cell dye; BioLegend). T cells were identified using CD3 (BV605; clone: UCHT1; BioLegend), CD4 (BUV395; clone: SK3; BD Biosciences), and CD8 (PerCpCy5.5; clone: RPA-T8; BioLegend). Non-T cells were identified using CD19 (BV711; clone: SJ25C1; BioLegend), CD14 (FITC; clone: M5E2; BD Biosciences), and HLA-DR (APC-Cy7; clone L243; BioLegend). Expression of Mucin-1 and Tetherin/BST-2 was measured on each cell subset using Mucin-1 (CD227; APC; clone 16A), Tetherin (CD317; PE; clone RS38E), and matched isotype controls (all BioLegend). Data were acquired on an LSRII flow cytometer (BD Biosciences), and flow cytometry data analysis was performed using FlowJo v10.0. Total PBMCs were identified based on forward and side scatter properties, with doublets (forward-scatter-height versus forward-scatter-width) subsequently excluded within viable (Aqua Viability Dye^−^) PBMCs. CD4^+^, CD8^+^, and CD4^−^CD8^−^ (double-negative) T cells were established within total CD3^+^ T cells (Fig. S4). Within CD3^−^ cells, B cells were identified as CD19^+^, monocytes as CD19^−^CD14^+^ and cell populations enriched for dendritic cells (DCs) as CD19^−^CD14^−^HLA-DR^+^ and enriched for NK/ILCs as CD19^−^CD14^−^HLA-DR^−^. Specific expression of Mucin-1 and tetherin on all cell populations was determined using isotype controls. Frequencies of CD4^+^ T cells at baseline were calculated as a percentage of viable PBMCs.

### Statistical analysis

Data visualization and statistical analyses were performed using GraphPad Prism v.8.4.2 (GraphPad, San Diego, CA, USA). Two-tailed paired Student’s *t*-tests were utilized, with each data point corresponding to a specific donor treated with various conditions. Comparisons between PWH and matched controls utilized unpaired two-tailed Student’s *t*-tests. Correlations were assessed by Spearman’s rank. Statistical significance was set at a threshold of *P* < 0.05.

## Data Availability

Next-generation sequencing data generated in this study have been deposited in the Sequence Read Archive under BioProjects PRJNA789251 (PBMC Interferomes) and PRJNA558974 (Clinical study PBMC). Fold-induction data of interferon-regulated genes and raw data sets used for correlation analyses can be found in Tables S1 to S6.

## SUPPLEMENTAL MATERIAL

Supplemental figures jvi.01089-26-s0001.docx Figures S1 to S3.

Supplemental tables jvi.01089-26-s0002.xlsx Tables S1 to S6.

ASM does not own the copyrights to Supplemental Material that may be linked to, or accessed through, an article. The authors have granted ASM a non-exclusive, world-wide license to publish the Supplemental Material files. Please contact the corresponding author directly for reuse.
