# Supplementary material for: Interferome profiling in the blood links IFNα5-Mucin-1 axis to HIV-1 pathogenesis
Source: J Virol. 2026 Sep 25;100(10):e01089-26. doi: 10.1128/jvi.01089-26 (PMC13712950; doi:10.1128/jvi.01089-26)
Supplement: Supplementary file 1 [file jvi.01089-26-s0001.docx]

**SUPPLEMENTARY FIGURES**

**Interferome Profiling in the Blood Links IFNα5-Mucin-1 Axis to HIV-1 Pathogenesis**

Kejun Guo^a,b^, Stephanie M. Dillon^a^, Emily C. Meneses^c^, Bradley S. Barrett^a^, Alexandra Norman^a^, Kaylee L. Mickens^a,d^, James H. Morrison^a^, Ulf Dittmer^e^, Kathrin Sutter^e,f^,

Eric M. Poeschla^a^, Katerina Kechris^c^, Cara C. Wilson^a,b,d^# and Mario L. Santiago^a,b,d^#

*^a^Division of Infectious Diseases, University of Colorado School of Medicine, Aurora, Colorado, USA*

*^b^RNA Bioscience Initiative, University of Colorado School of Medicine, Aurora, Colorado, USA*

*^c^Center for Innovative Design and Analysis, Department of Biostatistics and Informatics, University of Colorado School of Medicine, Aurora, Colorado, USA*

*^d^Department of Immunology and Microbiology, University of Colorado School of Medicine, Aurora, Colorado, USA*

*^e^Institute for Virology, University Hospital Essen, University of Duisburg-Essen, Essen, Germany*

*^f^Institute for the Research on HIV and AIDS-associated Diseases, University Hospital Essen, University of Duisburg-Essen, Essen, Germany*

#Address correspondence: Mario L. Santiago, mario.santiago@cuanschutz.edu, Cara C. Wilson, cara.wilson@cuanschutz.edu.

**Figure S1**

**Figure S1.** Normalization of PBMC interferome datasets. (A) Visualization of Trimmed Mean of M-Values (TMM) using edgeR. The Relative Log Expression Plots depict boxplot of deviations from gene medians. (B) Principal Component Analysis showing donor-dependence of transcriptome datasets from PBMC treated with various IFN-Is and mock. Colors correspond to donors in panel A.

**Figure S2**

**Figure S2.** Comparison of genes altered by diverse IFN-Is in PBMCs. The UpSet Diagram was constructed using UpSetR to visualize intersections between the genes altered by different IFN-Is in a matrix layout. IFN-regulated genes were defined based on an absolute fold-change >1.5 with an FDR cut-off of 20%.

**Figure S3**

**Figure S3.** Relaxed statistical thresholds for *MUC1* induction by diverse IFN-Is. (A) TMM-normalized *MUC1* expression levels were plotted for each 7 PBMC donors, showing that *MUC1* was induced, on average, 1.9-fold higher than the average of the other IFN-Is tested. Note that data from one donor in the IFNα5 treatment was 0. As this could be a technical issue with RNAseq, removal of this data point would increase the expression value from 7.6 to 8.9, with IFNα5 inducing *MUC1* 2.2-fold higher than the other IFN-Is tested. (B) IFNα5 induced *MUC1* to a more significant extent than the other IFN-Is, based on p-values and FDR. Lowering significance thresholds suggest that *MUC1* could also be induced by other IFN-Is to a lesser extent.
